# Integrative Analysis of Genome, 3D Genome, and Transcriptome Alterations of Clinical Lung Cancer Samples

**DOI:** 10.1016/j.gpb.2020.05.007

**Published:** 2021-06-08

**Authors:** Tingting Li, Ruifeng Li, Xuan Dong, Lin Shi, Miao Lin, Ting Peng, Pengze Wu, Yuting Liu, Xiaoting Li, Xuheng He, Xu Han, Bin Kang, Yinan Wang, Zhiheng Liu, Qing Chen, Yue Shen, Mingxiang Feng, Xiangdong Wang, Duojiao Wu, Jian Wang, Cheng Li

**Affiliations:** 1 Center for Bioinformatics, School of Life Sciences, Center for Statistical Science, Peking University, Beijing 100871, China; 2 BGI-Shenzhen, Shenzhen 518083, China; 3 Zhongshan Hospital Institute of Clinical Science, Fudan University, Shanghai Institute of Clinical Bioinformatics, Shanghai 200433, China; 4 Fudan University Center for Clinical Bioinformatics, Shanghai 200433, China; 5 Department of Thoracic Surgery, Zhongshan Hospital of Fudan University, Shanghai 200032, China; 6 School of Life Sciences, Tsinghua University, Beijing 100084, China; 7 BGI-Qingdao, Qingdao 266426, China; 8 Shenzhen Engineering Laboratory for Innovative Molecular Diagnostics, BGI-Shenzhen, Shenzhen 518083, China; 9 iCarbonX, Shenzhen 518053, China; 10 Digital Life Research Institute, Shenzhen 518110, China; 11 State Key Laboratory of Proteomics, National Center of Biomedical Analysis, Institute of Basic Medical Sciences, Beijing 100850, China; 12 China National GeneBank, BGI-Shenzhen, Shenzhen 518083, China

**Keywords:** Lung cancer, 3D genome, Copy number variation, Clinical sample, Integrative genomic analysis

## Abstract

Genomic studies of cancer cell alterations, such as mutations, **copy number variations** (CNVs), and translocations, greatly promote our understanding of the genesis and development of cancers. However, the **3D genome** architecture of cancers remains less studied due to the complexity of cancer genomes and technical difficulties. To explore the 3D genome structure in clinical **lung cancer**, we performed Hi-C experiments using paired normal and tumor cells harvested from patients with lung cancer, combining with RNA sequenceing analysis. We demonstrated the feasibility of studying 3D genome of clinical lung cancer samples with a small number of cells (1 × 10^4^), compared the genome architecture between **clinical samples** and cell lines of lung cancer, and identified conserved and changed spatial chromatin structures between normal and cancer samples. We also showed that Hi-C data can be used to infer CNVs and point mutations in cancer. By integrating those different types of cancer alterations, we showed significant associations between CNVs, 3D genome, and gene expression. We propose that 3D genome mediates the effects of cancer genomic alterations on gene expression through altering regulatory chromatin structures. Our study highlights the importance of analyzing 3D genomes of clinical cancer samples in addition to cancer cell lines and provides an **integrative genomic analysis** pipeline for future larger-scale studies in lung cancer and other cancers.

## Introduction

Lung cancer is the leading cause of cancer death [1], [2], of which lung adenocarcinoma (ADC) is the most common histological subtype. Genomic alterations in ADC such as point mutations, aneuploidy, copy number variations (CNVs), and DNA methylation have been comprehensively characterized to discover novel molecular subtypes, cancer-driving pathways, and therapeutic targets [3], [4]. Recently, the 3D genome structures have been studied with various experimental and computational methods [5], [6], [7], [8], [9], and the reorganization of spatial chromatin interactions in cancer cells is recognized as a new type of genomic alterations [10], [11]. For example, non-coding CNVs or mutations at topologically associated domain (TAD) boundaries or chromatin loop anchors result in *de novo* chromatin interactions and domains, which lead to activation of proto-oncogenes [12], [13], [14].

Most of the 3D cancer genome studies were performed on human cancer cell lines since a high number of cells (> 1 × 10^6^) are required for chromosome conformation capture experiments such as Hi-C and ChIA-PET to interrogate 3D genome interactions [6], [7], [15], [16], [17]. There is also a lack of the comparison between the 3D genomes of cancer cell lines and clinical cancer samples to confirm cancer cell lines as proper and accurate models to study the reorganization of 3D cancer genomes. Therefore, clinical applications of 3D genome techniques for patient samples and integration of 3D genome data with DNA sequencing and RNA sequencing (RNA-seq) data will better illuminate causes and consequences of cancer genome alterations [18].

In the present study, we explores the clinical application of 3D genome analyses by applying Hi-C to lung ADC samples and paired normal lung tissues using as few as 1 × 10^4^ cells. By integrating the 3D genome reorganization in lung cancer samples with CNVs, mutations, and gene expression changes in the same samples, we reveal the correlations between different data types and propose a model that 3D genome mediates functional consequences of genomic alterations in lung cancer.

## Results

### Improving Hi-C experiments with limited number of cells

**Figure 1:**
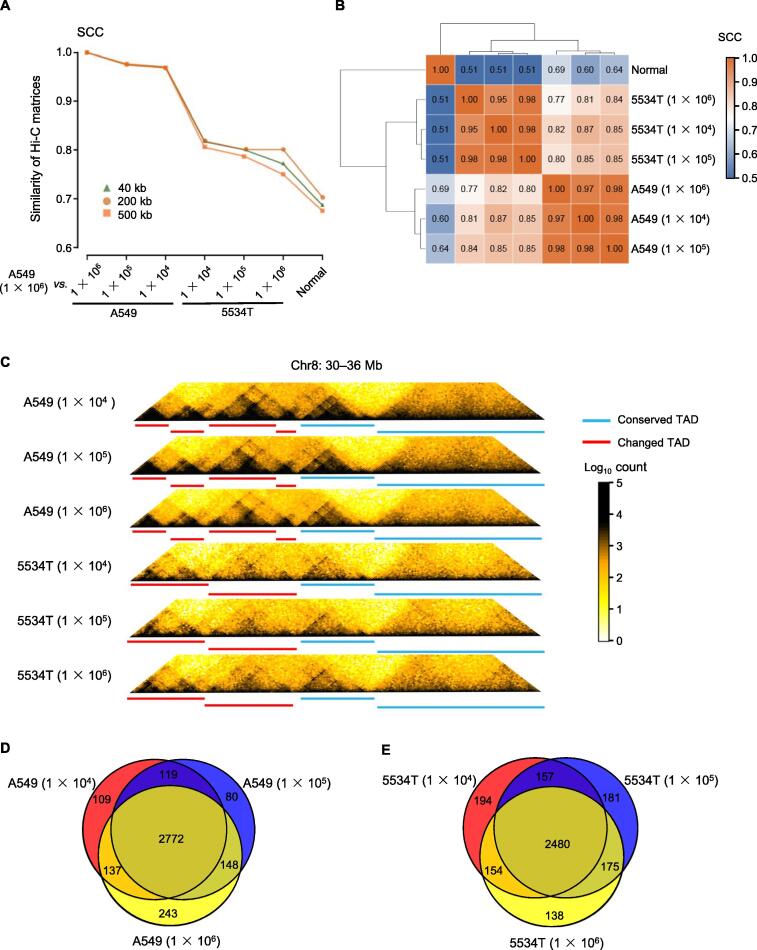
**Detecting the 3D genome of clinical lung cancer samples** A549 cell line and the 5534T cancer sample were used for *in situ* Hi-C at three cell number gradients. **A.** Plot showing SCC scores calculated by HiCRep among different Hi-C experiments. **B.** Correlations of Hi-C matrices between normal lung tissue (from Schmitt et al. [36]) and 5534T/A549 cells with different cell numbers calculated by HiCRep (resolution: 40 kb). **C.** Example of conserved and changed TADs in a region (chr8: 30–36 Mb) by comparing A549 and 5534T cells. **D.** and **E.** The number of conserved and changed TADs detected by Hi-C using different numbers of A549 cells (D) and 5534T cells (E). SCC, stratum-adjusted correlation coefficient; 5534T, a tumor sample collected from patient 5534; TAD, topologically associated domain.

Due to the low number of cells available in clinical lung cancer samples, we explored the viability of conducting *in situ* Hi-C experiments with fewer cells for the first time [6]. We performed Hi-C experiments with 1 × 10^4^, 1 × 10^5^, and 1 × 10^6^ cells from the A549 lung ADC cell line and a tumor sample collected from a patient (named 5534T). The raw interaction counts in the Hi-C data obtained with different cell numbers were highly correlated (Figure 1A and B, Figure S1). The normalized chromatin interactions and TADs identified with 1 × 10^4^ cells were highly similar to those identified using 1 × 10^5^ and 1 × 10^6^ cells, for both the A549 cell line and the 5534T tumor sample (Figure 1C). Specifically, 88% of the 3300 TADs identified using 1 × 10^6^ A549 cells were also found using 1 × 10^4^ cells, and 93% of the 3137 TADs identified using 1 × 10^4^ A549 cells were confirmed using 1 × 10^6^ cells (Figure 1D). Similar proportions were observed in the 5534T tumor sample (Figure 1E). These results demonstrate that 1 × 10^4^ cells are sufficient for the identification of TADs and chromatin interactions with high sensitivity and accuracy using Hi-C experiments.

### TADs and **chromatin** loops are altered significantly in clinical lung cancer samples

**Figure 2:**
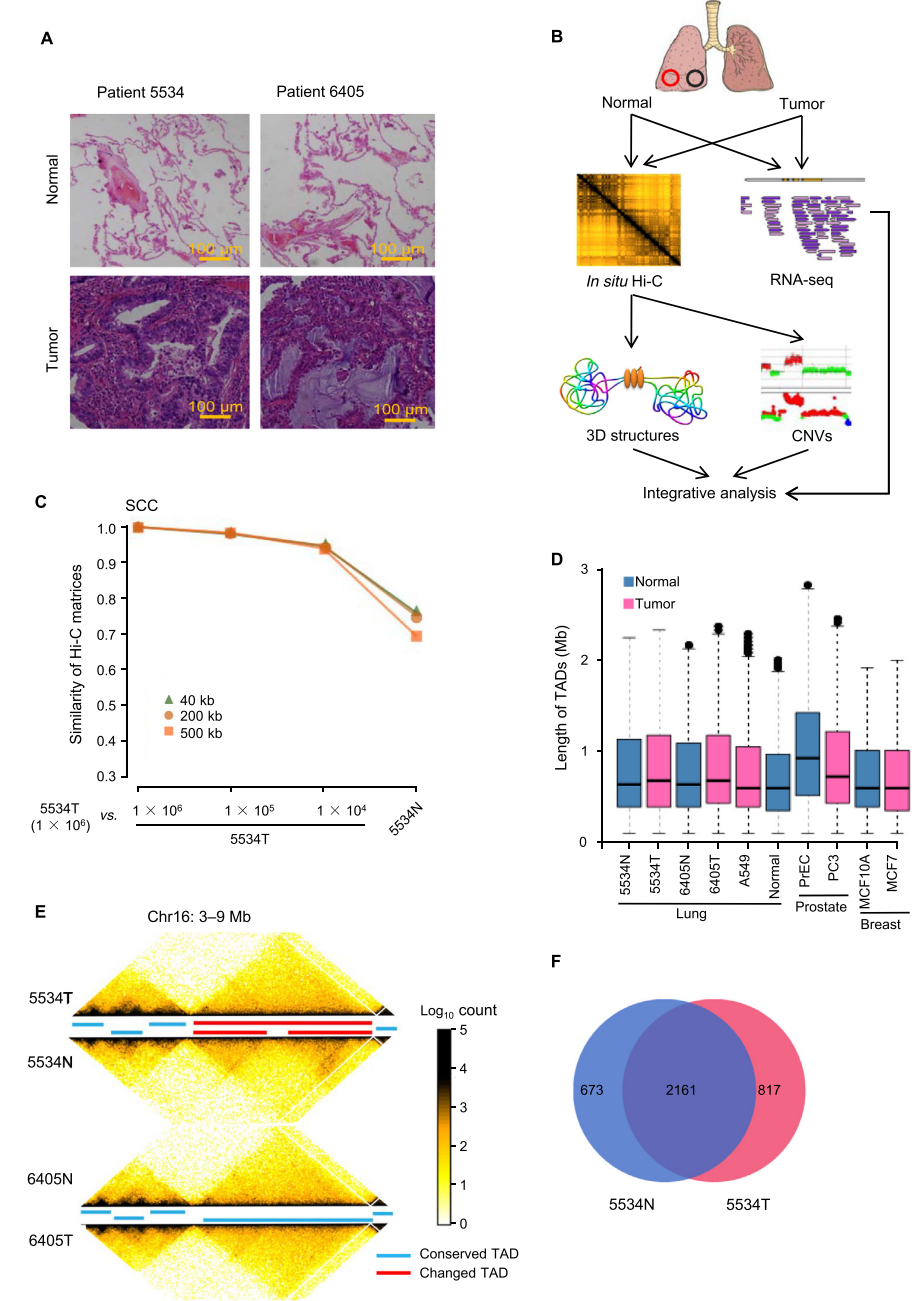
**3D genome structures of clinical normal and lung cancer samples** **A.** Histopathological images of normal and tumor clinical samples stained with hematoxylin and eosin. **B.** Outline of experiments and analyses in this study. **C.** Similarity of chromatin interactions evaluated by HiCRep in samples from patient 5534 at different resolutions. **D.** The length distribution of TADs in normal and cancer tissue samples as well as cell lines. **E.** Example of conserved and changed TADs in a region (chr16: 3–9 Mb) by comparing paired normal and tumor samples from lung cancer patients. **F.** The number of conserved and changed TADs between normal and tumor samples of patient 5534. CNV, copy number variation.

We next performed Hi-C and RNA-seq experiments on paired normal lung tissues and tumor samples from two lung ADC patients (patients 5534 and 6405; Figure 2A and B; Table 1, Table S1). Approximately 30%–40% of cells isolated from the tumor sample from patient 5534 were cancer cells, while the proportion of cancer cells was 10%–20% in the tumor sample from patient 6405 (Figures S2 and S3). The raw chromatin interaction matrices for the paired normal and tumor samples were largely correlated but showed noticeable differences (Figure 2C). We compared the raw Hi-C matrices between normal and cancer cell lines and primary tissues from the lung, prostate, and breast. The chromatin interactions among the samples from the lung and breast were highly similar and distinct from those in the samples from the prostate (Figure S4A and B), likely due to differences in the cell origins between the prostate cancer samples and the lung and breast cancer samples [19].

**Table 1:** **Patient information and characteristics**

**Patient ID**	**Age**	**Smoking status**	**Histological type**	**Differentiation**	**Tumor size (cm)**	**Lymph node metastasis**	**Pathological stage**	**Recurrence or metastasis**
5534	46	Non-smoker	ADC	Well	3	Yes	II	No
6405	62	Smoker	ADC	Moderate	2	No	I	No

*Note*: ADC, adenocarcinoma.

To more closely explore the 3D genome differences between normal and tumor tissues, we compared the TADs and A/B compartments [5], [20] derived from the chromatin interaction data across cell lines, tissue samples, and cancer types using caICB-normalized Hi-C data [21]. Unlike a previous study on prostate cancer cell lines [11], we did not observe a decrease in the overall length of TADs in our lung cancer tissue samples or in public Hi-C data for the MCF7 breast cancer cell line compared to their normal counterparts (Figure 2D). However, both conserved and altered TADs were detected in paired normal and tumor lung tissues (Figure 2E). Nearly 24% of TADs were altered in the lung tumor tissue sample from patient 5534 compared to its normal lung counterpart (Figure 2F), and similar differences in TADs were observed between normal and cancer cell lines from the breast and prostate (Figure S4C). These results imply that TAD alteration is a significant factor in lung cancer evolution since the alteration of TAD structures can lead to *de novo* interactions between enhancers and promoters [14], [22].

To identify the changes in chromatin loops, we applied the Fit-Hi-C [23] and HiCCUPS [6] methods to paired normal and tumor Hi-C data, and evaluated both shared and sample-specific chromatin loops in normal and tumor samples (Figure S5A and B). To confirm the accuracy of the identified loop interactions, aggregate peak analysis (APA) was performed and the enrichment heatmaps confirmed the identified loop interactions as well as the differences between the samples (Figure S5C and D). Similar to a previous study showing that TADs are more stable than chromatin loops [6], the loop interactions varied substantially between paired normal and tumor samples (Figure S5A and B).

### A/B compartments are largely conserved in normal and lung tumor samples

**Figure 3:**
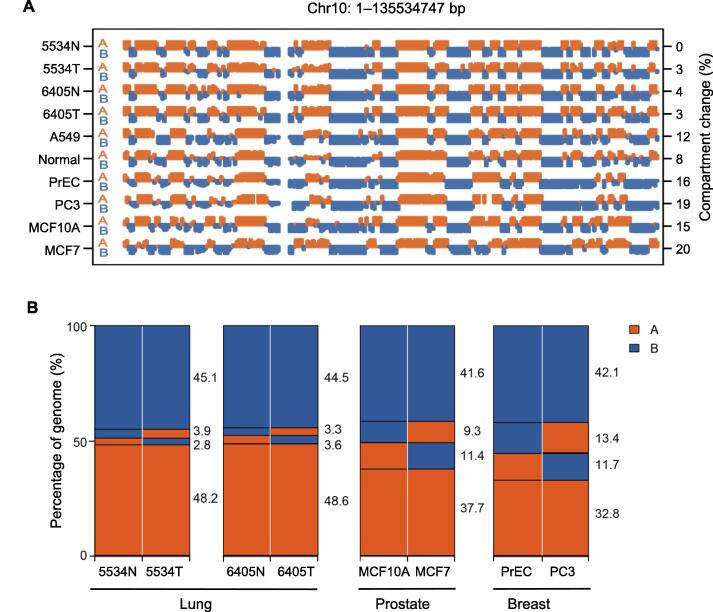
**A/B compartmentswitchingof different cancer cell lines and tissues** **A.** The A/B compartments of chromosome 10 inferred from Hi-C data of various samples. The percentage of chromosome 10 with A/B compartment switching in different samples was compared to 5534N. **B.** The percentage of genome with A/B compartment switching between paired normal and lung tumor tissues or between paired normal and cancer cell lines for breast and prostate.

In contrast to TADs, fewer differences in the frequency of A/B compartment changes were observed between normal and lung tumor samples than between different tissue types (Figure 3A). In the tumor sample from patient 5534, 3.9% of genomic regions changed from compartment B to A, while 2.8% changed from compartment A to B, compared to the compartments in the paired normal sample (Figure 3B). The corresponding values were 3.3% and 3.6% in the samples from patient 6405 (Figure 3B). These changes were smaller than those observed between paired normal and cancer cell lines for the prostate and breast (Figure 3B), possibly due to the different genetic backgrounds of the paired cell lines. The results suggest that genome alterations in lung cancer cells have a greater impact on smaller-scale factors such as TAD and loop structures, but have a less impact on larger-scale factors such as A/B compartments. Larger sample cohorts are needed to confirm these results.

### Successful CNV detection from Hi-C data

Hi-C interaction counts from cancer cells are influenced by CNVs and should be properly adjusted to obtain copy number-independent chromatin interactions [21]. This also implies that Hi-C reads can be analyzed to identify CNVs using an approach similar to that used for whole-genome sequencing (WGS) data. We used the HiCnv software [24] to obtain genome-wide CNVs from a previously published myeloma cell line (RPMI-8226) with Hi-C and WGS data (Figure S6A). The CNVs obtained from Hi-C were consistent with those obtained from WGS data (> 70% overlap, Figure S6B) and were not affected by the sequencing depth (Figure S6C).

**Figure 4:**
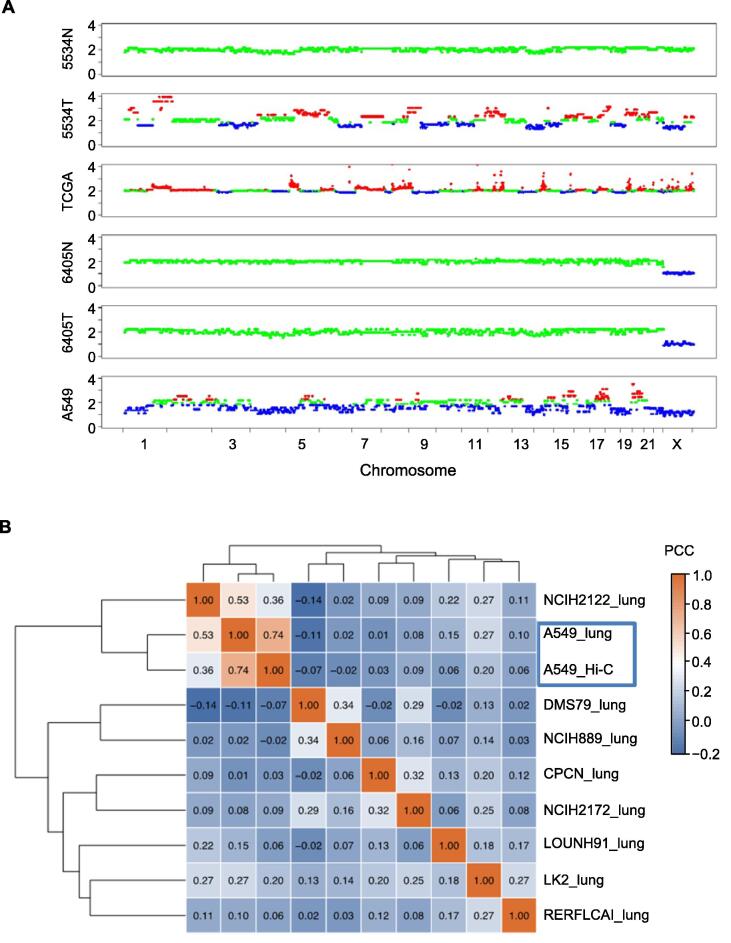
**Cancer CNVs identified from Hi-C data** **A.** Comparison of Hi-C-detected CNVs in lung cancer clinical samples and lung cancer cell line A549. Red represents copy number gain, blue represents copy number deletion, and green represents normal copy number. The “TCGA” row represents the average CNVs of 120 lung ADC patients from TCGA [3]. **B.** Correlation of CNVs of A549 cells detected from Hi-C data with the CNVs of various lung cancer cell lines detected by SNP microarrays in the CCLE database. TCGA, The Cancer Genome Atlas; ADC, adenocarcinoma; SNP, single nucleotide polymorphism; CCLE, Cancer Cell Line Encyclopedia; PCC, Pearson correlation coefficient.

We then identified genome-wide CNVs from the Hi-C data of A549 cells and lung samples (Figure 4A). Among the lung cancer cell lines in the Cancer Cell Line Encyclopedia (CCLE) database, CNVs of the A549 cell line detected using Hi-C showed the highest correlation with those of the A549 cell line detected using a single nucleotide polymorphism (SNP) array [25] (*P* < 0.01, Student’s *t*-test; Figure 4B, Figure S6D). CNVs detected in the 5534T lung tumor sample using Hi-C showed alteration patterns similar to those of typical CNVs in ADC samples available in The Cancer Genome Atlas (TCGA) [3], including copy number gains in chromosomes 1q, 7p, 8q, and 17q (Figure 4A). In contrast, the paired normal sample (5534N) showed no CNVs. We did not detect CNVs in the paired normal and tumor lung tissue samples from patient 6405, either because this patient had early-stage ADC (Table 1) or the tumor cell content in the sample was low.

### Successful mutation detection from Hi-C data and validation

**Figure 5:**
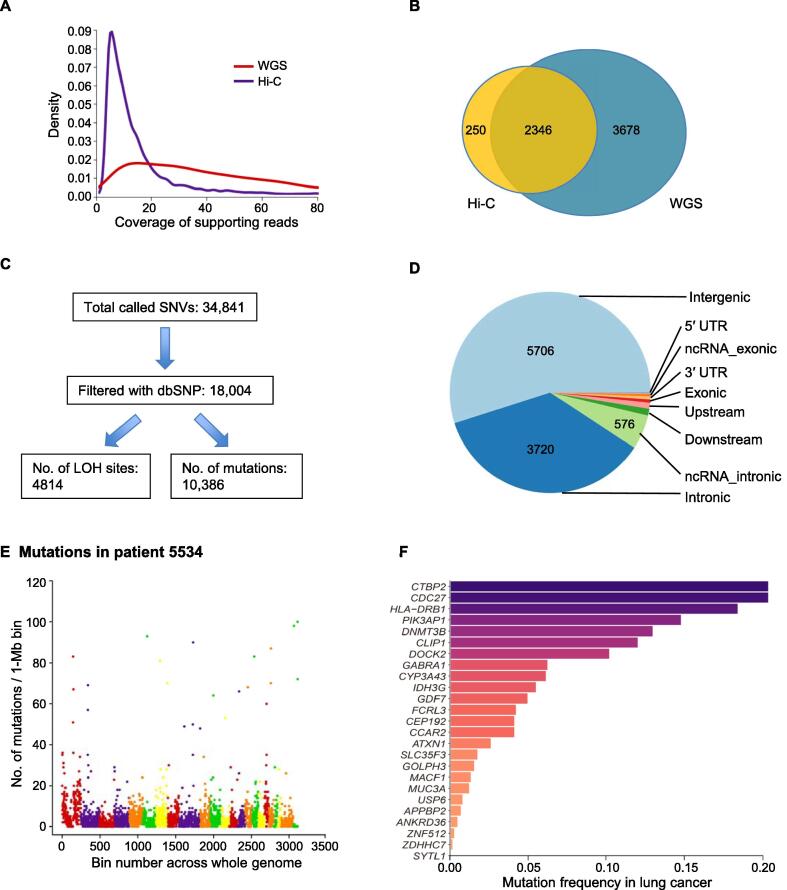
**Point mutations identified from tumor samples by Hi-C** **A.** Distribution of high-quality read depth of SNVs identified by WGS and Hi-C data in chromosome 22 of the U266 multiple myeloma cell line. **B.** Intersections of SNVs called by WGS and Hi-C data in chromosome 22 of U266 cells. **C.** Flowchart of the SNV calling and filtering to identify mutations from paired normal and lung tumor Hi-C data of patient 5534. **D.** Genome distribution of the mutations in patient 5534 called with Hi-C data. **E.** Mutation rates across the whole genome (chromesomes 1–22 and X) at the bin size of 1 Mb for patient 5534. **F.** Mutation-affected genes in lung cancer patient 5534 are prioritized based on their mutation frequencies in public lung cancer data sets using ANNOVAR-Phenolyzer. SNV, single nucleotide variation; WGS, whole-genome sequencing; LOH, loss of heterozygosity; UTR, untranslated region; ncRNA, non-coding RNA.

We next asked whether point mutations can be identified from the Hi-C data of cancer samples. Using previously generated WGS and Hi-C data for a multiple myeloma cell line U266 [17], we called single nucleotide variations (SNVs) for each data type using the same number of total reads (30×). The data were filtered for SNVs with at least three unique reads containing high-quality non-reference bases and differential variability (DV) ≥ 3. Taking chromosome 22 as an example, although the average read depth for the SNVs detected from Hi-C data was smaller than that detected from WGS data (Figure 5A), Hi-C detected 39% of the SNVs called from WGS data. Additionally, 90% of SNVs identified from Hi-C data were also detected from WGS data, demonstrating that the Hi-C-based SNV calls have moderate sensitivity and high precision (Figure 5B).

We hypothesized that the 10% of SNVs detected by Hi-C but not WGS were due to better sequence capture efficiency at genomic regions near restriction enzyme cutting sites in Hi-C experiments. The DV distribution showed that most of the SNVs uniquely detected by Hi-C had less than three reads in the WGS data (Figure S7A), indicating that the genomic regions containing these SNV sites were poorly captured by WGS experiments. In addition, the distances between SNVs and the nearest *Mbo*I cutting sites were significantly shorter for Hi-C-called SNVs than those for WGS-called SNVs (*P* < 0.01, Student’s *t*-test; Figure S7B), supporting the theory of a genome capture preference in Hi-C experiments. We further selected ten SNVs for validation by Sanger sequencing and obtained results for nine SNV sites (one site was not successfully sequenced because of PCR failures). Sanger sequencing showed that several SNVs uniquely detected by Hi-C were real variations of the reference sequence (Figure S7C).

We then called SNVs from the Hi-C data for primary lung tumor samples from patient 5534 and classified differences in SNVs between paired normal and tumor lung tissue samples as somatic mutations in cancer. In total, the paired Hi-C data for patient 5534 (Figure 5C) contained 10,386 mutations distributed across the entire genome (Figure 5D) and enriched in certain mutation hotspots (Figure 5E). Among them, 117 mutations affected the exons of 44 protein-coding genes and 33 mutations altered protein coding. We sorted these 44 genes using the ANNOVAR Web server according to the mutation frequencies in public lung cancer datasets (Figure 5F). Among the top genes in the list, *PIK3AP1* encodes a Toll-like receptor (TLR) signaling adapter crucial for linking TLRs to phosphoinositide-3-kinase (*PI3K*) activation and regulating tumor inflammatory responses [26]. Therefore, in addition to detecting 3D genomic structures, Hi-C can also detect genomic alterations such as CNVs and mutations in clinical samples of lung cancer.

### Integrating genome, 3D genome, and gene expression alterations in lung cancer

**Figure 6:**
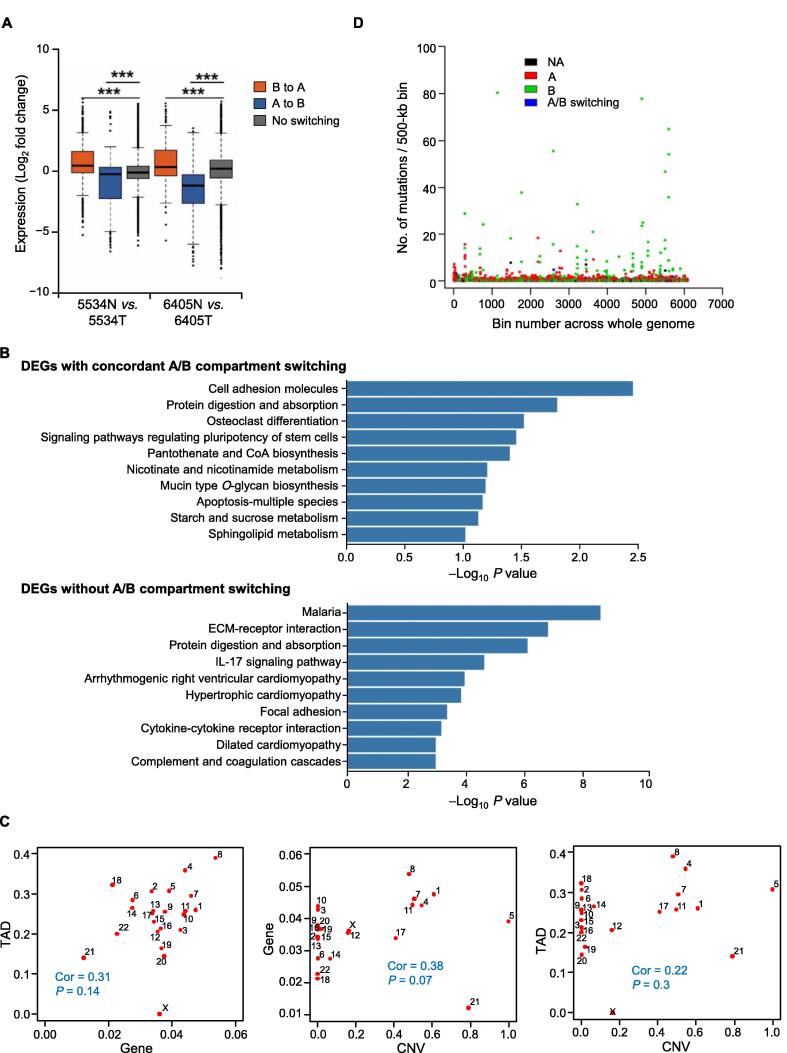
**Correlative analysis between genome, 3D genome, and gene expression changes** **A.** Boxplot showing expression changes of genes grouped by A/B compartment changes between normal and lung tumor samples. **B.** GO enrichment analysis for DEGs between normal and lung tumor samples of patient 5534 with concordant A/B compartment switching (top) or without A/B switching (bottom). **C.** Scatter plots showing the proportion of chromosome (or genes in a chromosome) that have altered CNVs, TADs, or DEGs in patient 5534. The chromosomes are indicated by numbers in the plot. **D.** Mutation rates across the whole genome (chromesomes 1–22 and X) at the bin size of 500 kb for patient 5534, colored by these bins’ A/B compartment states. GO, Gene Ontology; DEG, differentially expressed gene.

To explore whether the 3D genome mediates the effect of genomic alterations on gene expression in lung cancer, we investigated the correlations between CNVs, mutations, and 3D genomic structures detected using Hi-C and the transcriptome detected using RNA-seq in the same samples. Switches between compartments A and B were associated with changes in gene expression (Figure 6A). Genes that changed from compartment B to A were up-regulated, while those that changed from compartment A to B were down-regulated, consistent with the findings in breast cancer [10]. Among the differentially expressed genes (DEGs) between paired normal and tumor samples from patient 5534, 5% of DEGs were located in genomic regions with concordant A/B compartment switching and 92% were in genomic regions without compartment switching. This suggests that most expression dysregulations in cancer are due to *trans*-regulatory mechanisms. Notably, cell adhesion pathways were enriched in both gene groups (Figure 6B), suggesting that the dysregulation of specific pathways in lung cancer is likely related to 3D genomic alterations.

We next assessed the correlations between chromosome-wise alterations in gene expression, copy numbers, and 3D genomic structures. In the 5534T tumor sample, chromosomes in which more regions were affected by CNVs contained more changes in TADs and dysregulated genes (Figure 6C). In addition, mutation hotspots in the 5534T tumor sample occurred primarily in constitutive compartment B in both the normal and tumor samples (Figure 6D), consistent with the known associations between repressive chromatin regions and higher mutation frequencies [27].

## Discussion

The exploration of 3D genome architectures provides fundamental insights into key cellular processes such as DNA replication [28], [29] and gene regulation [30], [31], but there have been few studies on 3D cancer genomes using clinical cancer samples. In the present study, we attempted to resolve the issue of performing 3D genome experiments using a limited number of cells from clinical samples and analyzing cancer multi-omics data. We demonstrated that 1 × 10^4^ cells are sufficient for *in situ* Hi-C experiments and for obtaining spatial structure information, including TADs and A/B compartments in both cancer cell lines and clinical samples. We illustrated the feasibility of identifying CNVs and point mutations from Hi-C data for tumor samples. CNVs identified by Hi-C showed good concordance with those identified by WGS, and Sanger sequencing confirmed that Hi-C can better identify SNVs in chromosomal regions with more restriction enzyme cutting sites. These results provide a cost-effective solution for obtaining mutation, SNV, and 3D genome information using only Hi-C experiments. Notably, mutation detection with Hi-C has not been explored previously and is worth further optimization both experimentally and analytically.

Previous studies on 3D cancer genomes mostly utilized cancer cell lines. In this study, we showed that the 3D genome structure of primary lung cancer cells from ADC patients is significantly different from that of lung cancer cell lines. TADs identified in paired normal and tumor lung samples had similar length distributions and differed from previous findings in prostate cancer cell lines [11]. Moreover, the switching frequency between compartments A and B was much lower between paired normal and tumor samples from the same patient (~ 6%) than between normal and cancer cell lines with different genetic backgrounds (20%–25%). These findings highlight the importance of studying 3D genome architecture using primary lung cancer samples to confirm the findings obtained with cancer cell lines.

**Figure 7:**
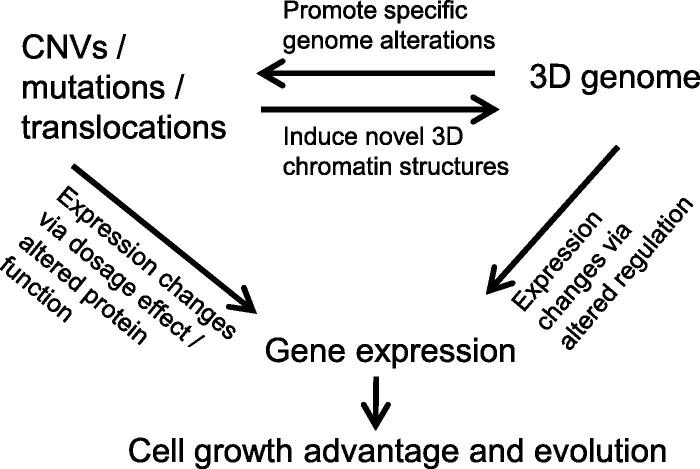
**A model for how the 3D genome mediates the effect of genome alterations on transcriptome**

Integrating 3D genome and gene expression information for paired normal and tumor samples can yield clues regarding the pathways and mechanisms that drive cancer evolution. We identified a set of DEGs with concordant A/B compartment switching, which enriched genes with cell adhesion functions including *CEACAM1* and Osteopontin (*OPN*). The *CEACAM* gene family belongs to the immunoglobulin superfamily, which contains 12 members in humans, and *CEACAM1* is an independent prognostic factor in ADC patients who undergo surgery [32]. The overexpression of *OPN* is associated with more aggressive phenotypes in human non-small cell lung cancer (NSCLC) [33]. Therefore, specific genes and pathways involving lung cancer development may be associated with 3D genomic alterations. We also demonstrated significant correlations between the copy numbers, 3D genome, and gene expression alterations, supporting the theory that certain cancer genomic alterations impact gene expression through the alteration of 3D genomic structures [11], [12] (Figure 7). This model also incorporates previous findings that 3D genome structures in normal cells induce specific chromosomal translocations in cancer [34], [35].

In conclusion, we performed a pioneering 3D genome study using paired normal and tumor samples from clinical lung cancer patients. We showed that Hi-C data can be used to discover cancer CNVs and mutations and provide multiple types of information regarding the genome and 3D genomic alterations. Our study highlights the importance of analyzing the 3D genome of clinical cancer samples and comparing the results with findings from cancer cell lines. The results also provide analysis workflows for future larger-scale 3D genome studies of cancer samples. The correlative findings require experimental validation such as genome-editing to confirm potential causative relationships among genome alterations, 3D genome, and gene expression dysregulation in cancer.

## Materials and methods

### Cell line and human samples

The human NSCLC cell line A549 was acquired from American Type Culture Collection (ATCC). Tumor and adjacent normal samples were collected from two adjuvant chemotherapy-naïve patients with lung ADC at the Zhongshan Hospital of Fudan University (Shanghai, China). Information about patient characteristics is summarized in Table 1. The adjacent normal tissue refers to the tissue that locates away from the tumor more than 5 cm in the lobectomy specimen, which is also confirmed by hematoxylin and eosin staining.

The Hi-C data used in this study included: 1) 5534N/T and 6405N/T (paired normal and tumor lung tissues from this study); 2) A549 (lung cancer cell line from this study); 3) Normal (normal lung tissue data from Schmitt et al. [36]); 4) PrEC (normal prostate epithelial cell line data from Taberlay et al. [11]); 5) PC3 (prostate cancer cell line data from Taberlay et al. [11]); 6) MCF-10A (mammary epithelial cell line data from Barutcu et al. [10]); 7) MCF-7 (breast cancer cell line data from Barutcu et al. [10]); and 8) GM12878 (normal B cell data from Rao et al. [6]).

### Cell preparation

For cell lines, A549 cells were maintained in RPMI-1640 (Catalog No. 11875, Life Technologies, Carlsbad, CA) supplemented with 10% fetal bovine serum (FBS) at 37 °C in a humidified atmosphere containing 5% CO_2_. Cells were cultured to about 80% confluence, and digested by trypsin (Catalog No. 25300054, ThermoFisher Scientific, Waltham, MA). Detached cells were centrifuged at 300 *g* for 5 min, and a final pellet was obtained. For human samples, tissues were harvested into a tissue culture dish, and washed with phosphate-buffered saline (PBS) for several times. A small part was cut and saved into RNAlater RNA stabilization solution at −80 °C for RNA-seq. Tissue left were minced into pieces of 1–2 mm with sterile scissors, and transferred into a 15-ml conical tube. After 10 ml of collagenase II solution (0.5 mg/ml in PBS) was added, it was incubated at room temperature on a shaker for 30 min. And then cell suspension was filtered through a 70-µm cell strainer to eliminate clumps and debris. Cells were collected in a conical tube and centrifuged at 1100 r/min for 10 min at room temperature. The pellet was resuspended in BD lysing buffer and incubated 5–10 min at room temperature to lyse red blood cells. Cell suspension was collected and centrifuged at room temperature as above. The pellet was resuspended in PBS and filtered through a 40-µm cell strainer. Cells were centrifuged and then the final pellet was obtained. The final pellets of both A549 cell line and human samples were resuspended respectively in fresh PBS. Cell count was performed. And then cell suspension of a final concentration of 1 × 10^6^ cells per ml in PBS was prepared. The following Hi-C crosslinking experiment was performed immediately.

### Hi-C libraries

The Hi-C experiments were performed as previously described [6] to generate Hi-C libraries derived from A549 cell line and human lung tissues. Cross-linked cells of the A549 cell line and human sample 5504T were divided into three groups, at cell numbers of 1 × 10^6^, 1 × 10^5^, and 1 × 10^4^, to test the quality of Hi-C library of various cell numbers.

### RNA-seq experiments and analyses

mRNA extraction and library construction were performed following the user’s instructions (Catalog No. E7645, NEB, Beverly, MA). Each sample had two biological repeats and at least 20 million paired-end reads were sequenced for each repeat. TopHat2 was used for read mapping (hg19) and Cufflinks for quantifying gene expression [37]. DESeq2 [38] was used for the downstream analyses.

### Hi-C data analysis

Read mapping and filtering of the Hi-C data were performed following previous methods [6]. First, reads were aligned to the human reference genome (hg19) with Bowtie2, and low mapping quality reads (MAPQ < 10) and PCR duplicates were removed separately by SAMtools and Picard tools. Then, we used the filtered contacts to create chromatin contact maps at different resolutions (40 kb, 500 kb) by HiC-Pro. We utilized a linear regression-based chromosome-level adjustment method called caICB to normalize raw interaction matrices [21]. The correlation between raw interaction count matrices of Hi-C samples was evaluated by HiCRep [39].

### A/B compartment analysis

We used caICB-normalized interaction matrices at 500-kb resolution to detect chromatin compartment types by R-package HiTC [40]. By doing principal component analysis (PCA), we segregated all chromosomal bins into two parts according to signs of PC1. Then the bins with higher overall gene density were assigned as A compartments, and the other bins were assigned as B compartments.

### TAD analysis

We used caICB-normalized interaction matrices at 40-kb resolution to call TADs by a Perl script matrix2insulation.pl (https://github.com/blajoie/crane-nature-2015) [41]. Then we converted adjacent TAD boundaries to corresponding TADs, and TADs were filtered through the following steps. First, only TADs with a length larger than 200 kb were kept. Second, TADs located in telomeres or centromeres were removed. We used BEDtools (intersectBed -f 0.80 -r) to identify conserved TADs that have more than 80% overlapping regions between two samples.

### Loop analysis

For the HiCCUPS method, Juicer Tools Pre was used to create the 40-kb normalized Hi-C contact matrix (.hic file) based on the allValidPairs files from HiC-Pro. Then we used Juicer Tools hiccups (-m 512 -r 40000 -k KR -f 0.1 -p 1 -i 3 -t 0.02,1.5,1.75,2 -d 80000) to call loops. For Fit-Hi-C method, we used the script in HiC-Pro to transform the 40-kb normalized Hi-C contact result matrix to a raw interaction count file and a bias file calculated by ICE. Then, Fit-Hi-C was done with default parameters. Finally, significant interactions were selected with *q*-value < 0.01.

### Calling CNVs and point mutations from Hi-C data

CNVs were called by the HiCnv software at 40-kb resolution with the “*.bwt2merged.bam” files from the output of HiC-Pro. We filtered out restriction enzyme fragments with GC content < 0.2 and mappability < 0.5 as HiCnv recommended and called CNVs separately for normal and tumor samples. SNVs were called by SAMtools/BCFtools (samtools mpileup -q10 -t DP,DV -f ref.fa sample.bam | bcftools call -vm), filtered with arguments (DP > 8, DV > 4) and dbSNP Ver. 146, followed with ANNOVAR annotation [42].

### **Gene Ontology** enrichment analysis

We used the DAVID (https://david.ncifcrf.gov) Bioinformatics Resources 6.7 for Gene Ontology (GO) enrichment analysis [43]. All human genes were used as the background gene list.

## Ethical statement

Informed written consents were obtained from the human subjects involved in this study with approval by the institutional ethical committee, and the research protocol was approved by the Ethical Evaluation Committee of Zhongshan Hospital of Fudan University, China.

## Code availability

All analysis codes and reproducible Rmd files related to this work are available at GitHub (https://github.com/ChengLiLab/LungCancerHi-C).

## Data availability

The raw sequencing data generated by this project are deposited in the Genome Sequence Archive [44] at the National Genomics Data Center, Beijing Institute of Genomics, Chinese Academy of Sciences / China National Center for Bioinformation (GSA: CRA000173 with BioProject: PRJCA000333) which are publicly accessible at https://ngdc.cncb.ac.cn/gsa, and in the CNGB Sequence Archive (CNGBdb: CNP0000704) which are publicly accessible at https://db.cngb.org/cnsa/.

## CRediT author statement

**Tingting Li:** Resources, Formal analysis, Writing - original draft, Writing - review & editing. **Ruifeng Li:** Formal analysis, Resources, Writing - original draft, Writing - review & editing. **Xuan Dong:** Resources, Writing - original draft. **Lin Shi:** Resources. **Miao Lin:** Resources. **Ting Peng:** Formal analysis, Writing - review & editing. **Pengze Wu:** Formal analysis. **Yuting Liu:** Formal analysis. **Xiaoting Li:** Formal analysis. **Xuheng He:** Resources. **Xu Han:** Resources. **Bin Kang:** Resources. **Yinan Wang:** Formal analysis. **Zhiheng Liu:** Formal analysis. **Qing Chen:** Formal analysis. **Yue Shen:** Supervision. **Mingxiang Feng:** Resources. **Xiangdong Wang:** Conceptualization, Supervision. **Duojiao Wu:** Conceptualization, Supervision. **Jian Wang:** Conceptualization, Supervision. **Cheng Li:** Conceptualization, Supervision, Writing - original draft, Writing - review & editing, Project administration. All authors have read and approved the final manuscript.

## Competing interests

The authors have declared no competinginterests.

## Supplementary material

Supplementary Figure S1 **Correlation heatmaps between samples based on Hi-C matrices with different resolution**. **A**. and **B**. SCC scores between different Hi-C experiments are calculated with interaction matrices at 200kb resolution (**A**) or 500kb resolution (**B**).

Supplementary Figure S2 **Estimated tumor cell content of two patient samples based on Hi-C data**. The x-axis represents the scaled read counts in tumor segments, the y-axis represents the allelic ratios of reads supporting the reference allele evaluated at germline heterozygous positions in those segments. The white does are actual segments and red lines are the integer copy-number states.

Supplementary Figure S3 **Flow cytometry experiment of five lung cancer patients**. Peri-tumor and tumor samples were first treated as described in Methods and then isolated with specific antibodies by flow cytometry. The y-axis represents the percentage of cells isolated with given antibody. CD3, CD8: markers of T cells; E-cadherin, CD66: markers of epithelial cells; CD11c, marker of dendritic cells; PDGFRa: markers of fibroblast cells.

Supplementary Figure S4 **Chromatin interactions in different cancer cell lines and tissues**. **A**. Example Hi-C chromatin interaction matrices of normal and cancer samples. See Methods for sample description. **B**. Hierarchical clustering of normal and cancer samples based on the pairwise SCC similarity calculated using chromatin interaction matrices of chromosome 14. **C**. The number of conserved and changed TADs between paired normal and tumor cell lines for breast cancer (left) and prostate cancer (right).

Supplementary Figure S5 **Chromatin loop interactions identified from Hi-C data**. **A.** and **B.** The number of overlapping and specific chromatin loops between samples identified by Fit-HiC (A) or HiCCUPUS (B). **C**. Aggregation peak analysis of chromatin loops identified by HiCCUPUS. **D**. Interaction heatmaps of a 1.2 Mb region on chromosome 1 (left) and chromosome 4 (right) of sample 6405N (blue, top right) and 6405T (red, bottom left). One normal specific loop (chr1:78.16-78.2 Mb with chr1:78.4-78.44 Mb) and one tumor specific loop (chr4:110.32-110.36 Mb with chr4:110.72-110.76 Mb) are highlighted.

Supplementary Figure S6 **CNVs identified from Hi-C data**. **A.** CNVs of RPMI-8226 cell line identified from WGS or Hi-C data (adapted from [17]). **B**. The common and specific CNVs detected from WGS and Hi-C data of RPMI-8226. **C**. The X-axis represents a sampled portion of the sequencing data from the full Hi-C sequencing data. Y-axis is the percentage of CNVs detected from a sampled Hi-C data set as compared to CNVs detected from full Hi-C data set. **D**. Comparison of A549 CNVs detected from Hi-C and the CCLE database. The X-axis is the inferred copy number from Hi-C data. The Y axis is the CNV values from CCLE. The heatmap shows the number of chromosomal regions that have respective CNV values from the two methods.

Supplementary Figure S7 **Characteristics and validation of Hi-C detected SNVs**. **A**. The distribution of DVs in WGS or Hi-C data for SNVs only identified by Hi-C. WGS: whole genome sequencing, DV: number of high-quality reads with non-reference bases. **B**. The distribution of distances between all WGS or Hi-C identified SNVs to their nearest MboI cutting sites. **C**. Validation of randomly selected 10 SNV sites by Sanger sequencing.

Supplementary Table S1 **Summary of the Hi-C read numbers**.
