# Supplementary figures and images for: Integrative Analysis of Genome, 3D Genome, and Transcriptome Alterations of Clinical Lung Cancer Samples
Source: Genomics Proteomics Bioinformatics. 2021 Jun 8;19(5):741–53. doi: 10.1016/j.gpb.2020.05.007 (PMC9170781; doi:10.1016/j.gpb.2020.05.007)

## Slide 1
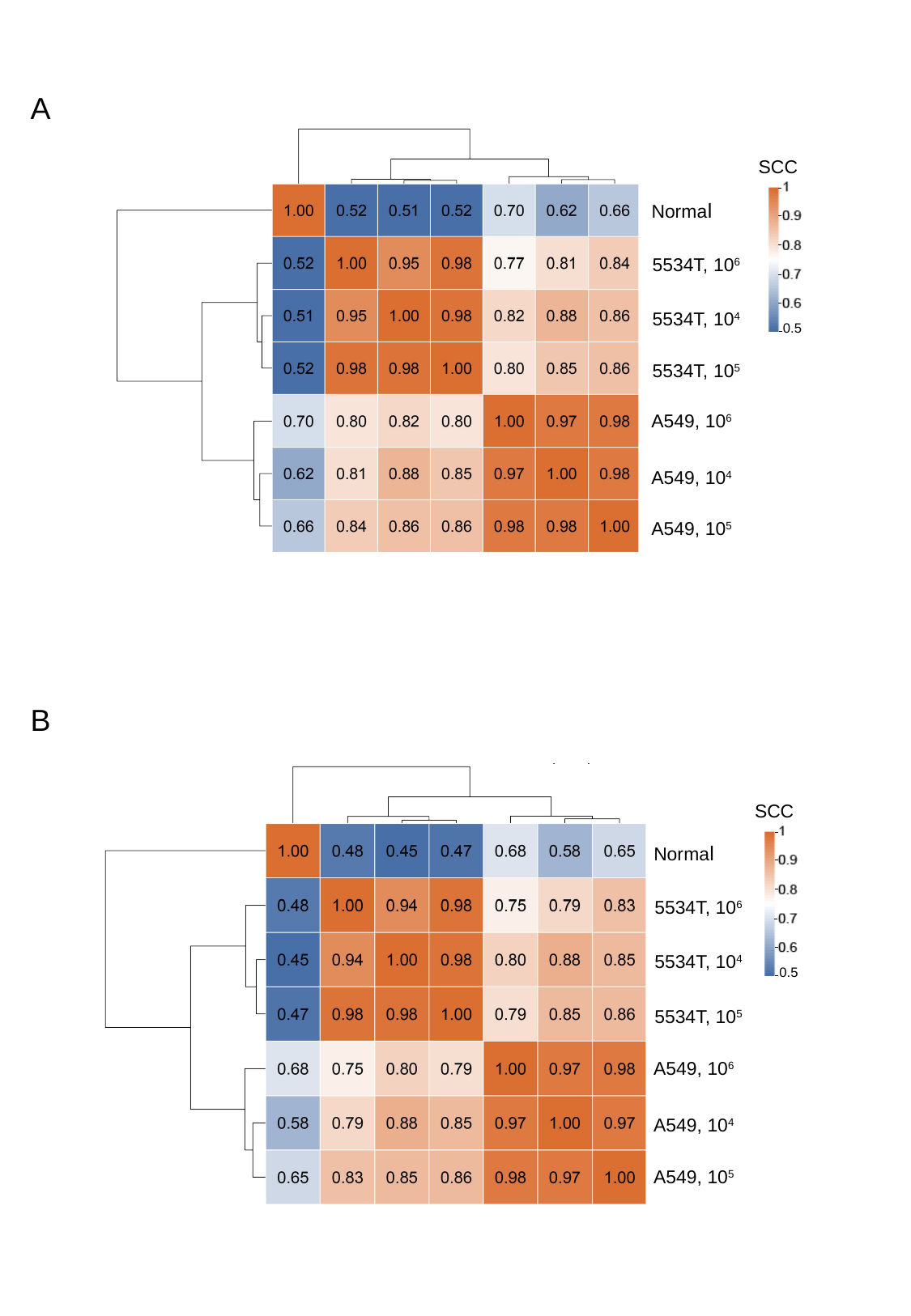

A
SCC
0.5
Normal
5534T, 106
5534T, 104
5534T, 105
A549, 106
A549, 104
A549, 105
B
SCC
0.5
Normal
5534T, 106
5534T, 104
5534T, 105
A549, 106
A549, 104
A549, 105

Supplement: Supplementary Figure S1 — Correlation heatmaps between samples based on Hi-C matrices with different resolution. A. and B. SCC scores between different Hi-C experiments are calculated with interaction matrices at 200kb resolution (A) or 500kb resolution (B). [file mmc1.pptx]

## Slide 1
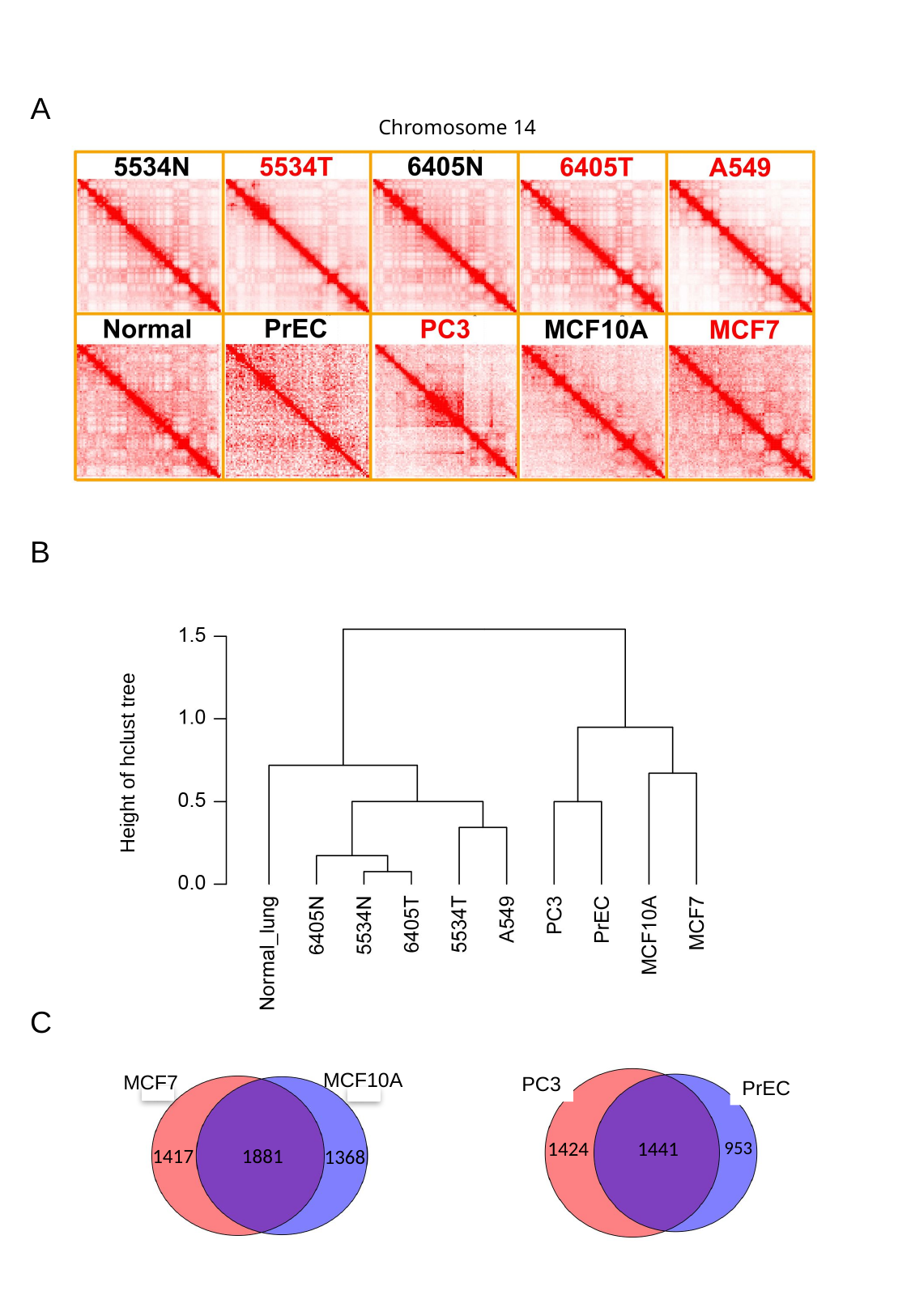

A
Chromosome 14
B
Height of hclust tree
C
PC3
PrEC
1424
1441
953
MCF10A
MCF7
1417
1881
1368

Supplement: Supplementary Figure S4 — Chromatin interactions in different cancer cell lines and tissues. A. Example Hi-C chromatin interaction matrices of normal and cancer samples. See Methods for sample description. B. Hierarchical clustering of normal and cancer samples based on the pairwise SCC similarity calculated using chromatin interaction matrices of chromosome 14. C. The number of conserved and changed TADs between paired normal and tumor cell lines for breast cancer (left) and prostate cancer (right). [file mmc4.pptx]

## Slide 1
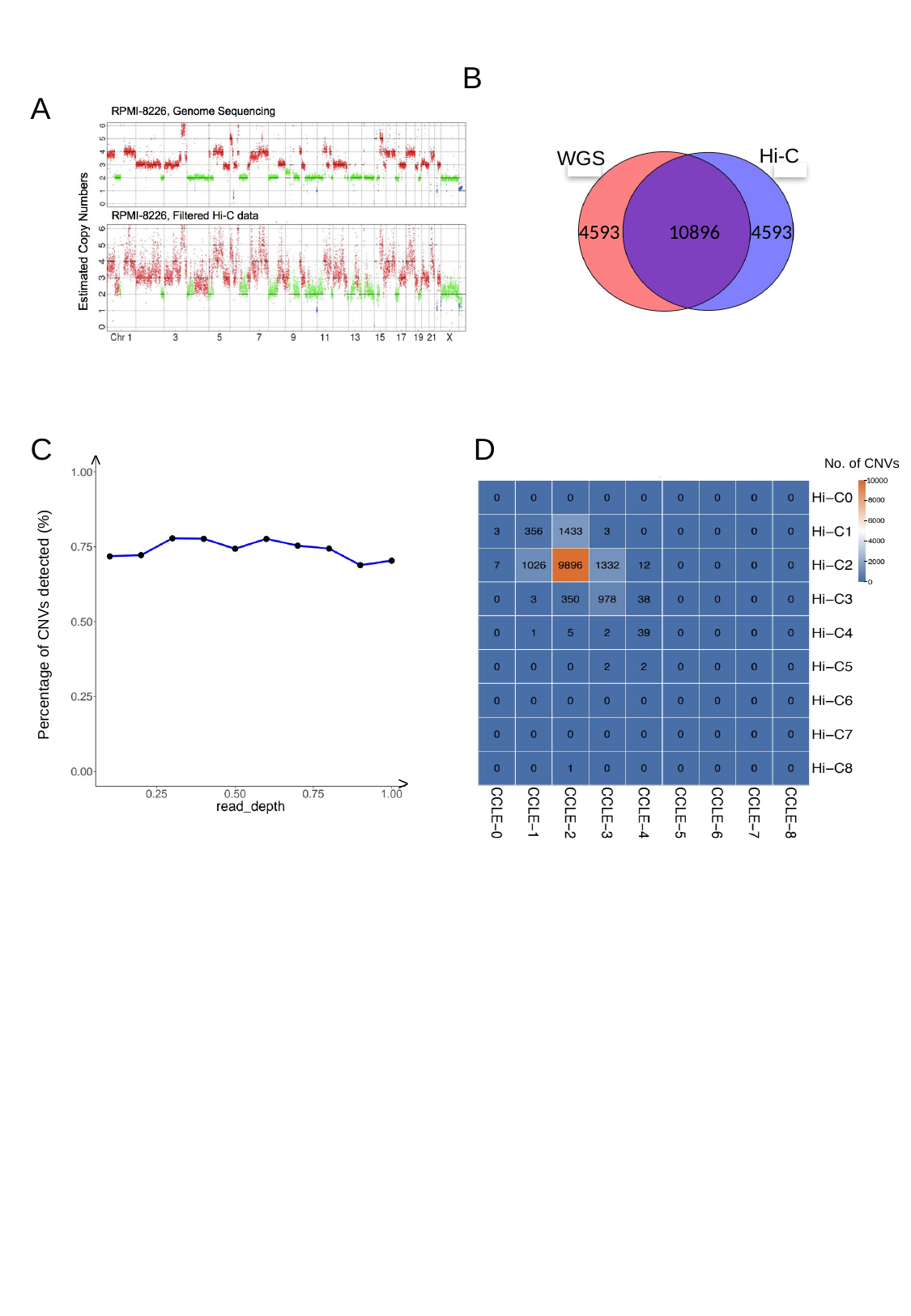

B
A
Hi-C
WGS
4593
4593
10896
C
D
>
>
No. of CNVs
10000
Percentage of CNVs detected (%)

Supplement: Supplementary Figure S6 — CNVs identified from Hi-C data. A. CNVs of RPMI-8226 cell line identified from WGS or Hi-C data (adapted from [17]). B. The common and specific CNVs detected from WGS and Hi-C data of RPMI-8226. C. The X-axis represents a sampled portion of the sequencing data from the full Hi-C sequencing data. Y-axis is the percentage of CNVs detected from a sampled Hi-C data set as compared to CNVs detected from full Hi-C data set. D. Comparison of A549 CNVs detected from Hi-C and the CCLE database. The X-axis is the inferred copy number from Hi-C data. The Y axis is the CNV values from CCLE. The heatmap shows the number of chromosomal regions that have respective CNV values from the two methods. [file mmc6.pptx]

## Slide 1
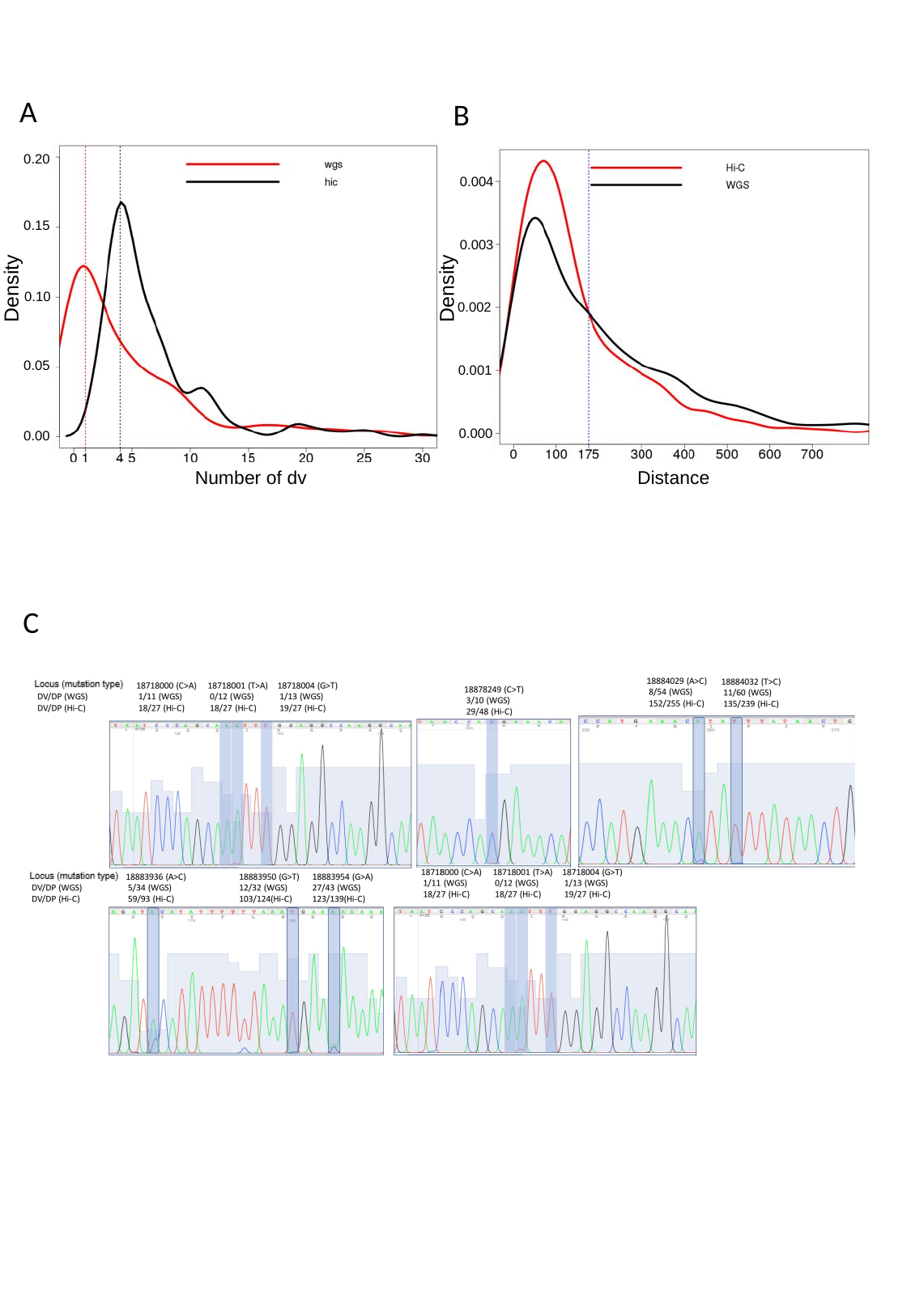

A
B
0.20
0.004
0.15
0.003
Density
Density
0.10
0.002
0.05
0.001
0.000
0.00
Number of dv
Distance
C

Supplement: Supplementary Figure S7 — Characteristics and validation of Hi-C detected SNVs. A. The distribution of DVs in WGS or Hi-C data for SNVs only identified by Hi-C. WGS: whole genome sequencing, DV: number of high-quality reads with non-reference bases. B. The distribution of distances between all WGS or Hi-C identified SNVs to their nearest MboI cutting sites. C. Validation of randomly selected 10 SNV sites by Sanger sequencing. [file mmc7.pptx]
