# Supplementary material for: Integrative Analysis of Genome, 3D Genome, and Transcriptome Alterations of Clinical Lung Cancer Samples
Source: Genomics Proteomics Bioinformatics. 2021 Jun 8;19(5):741–53. doi: 10.1016/j.gpb.2020.05.007 (PMC9170781; doi:10.1016/j.gpb.2020.05.007)
Supplement: Supplementary Figure S2 — Estimated tumor cell content of two patient samples based on Hi-C data. The x-axis represents the scaled read counts in tumor segments, the y-axis represents the allelic ratios of reads supporting the reference allele evaluated at germline heterozygous positions in those segments. The white does are actual segments and red lines are the integer copy-number states. [file mmc2.pptx]

## Slide 1
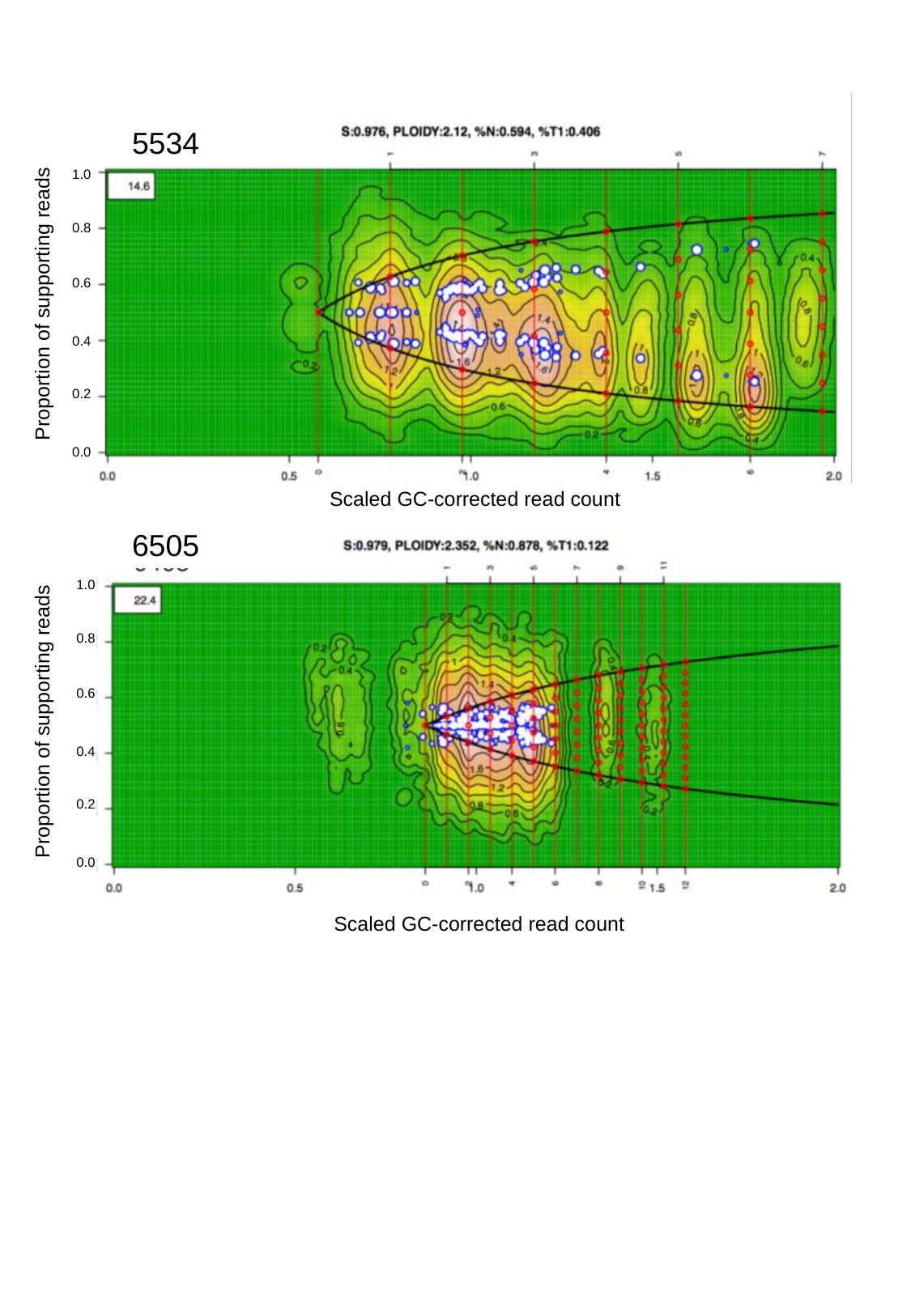

5534
1.0
0.8
0.6
Proportion of supporting reads
0.4
0.2
0.0
Scaled GC-corrected read count
6505
1.0
0.8
0.6
Proportion of supporting reads
0.4
0.2
0.0
Scaled GC-corrected read count
