# Supplementary material for: Integrative Analysis of Genome, 3D Genome, and Transcriptome Alterations of Clinical Lung Cancer Samples
Source: Genomics Proteomics Bioinformatics. 2021 Jun 8;19(5):741–53. doi: 10.1016/j.gpb.2020.05.007 (PMC9170781; doi:10.1016/j.gpb.2020.05.007)
Supplement: Supplementary Figure S3 — Flow cytometry experiment of five lung cancer patients. Peri-tumor and tumor samples were first treated as described in Methods and then isolated with specific antibodies by flow cytometry. The y-axis represents the percentage of cells isolated with given antibody. CD3, CD8: markers of T cells; E-cadherin, CD66: markers of epithelial cells; CD11c, marker of dendritic cells; PDGFRa: markers of fibroblast cells. [file mmc3.pptx]

## Slide 1
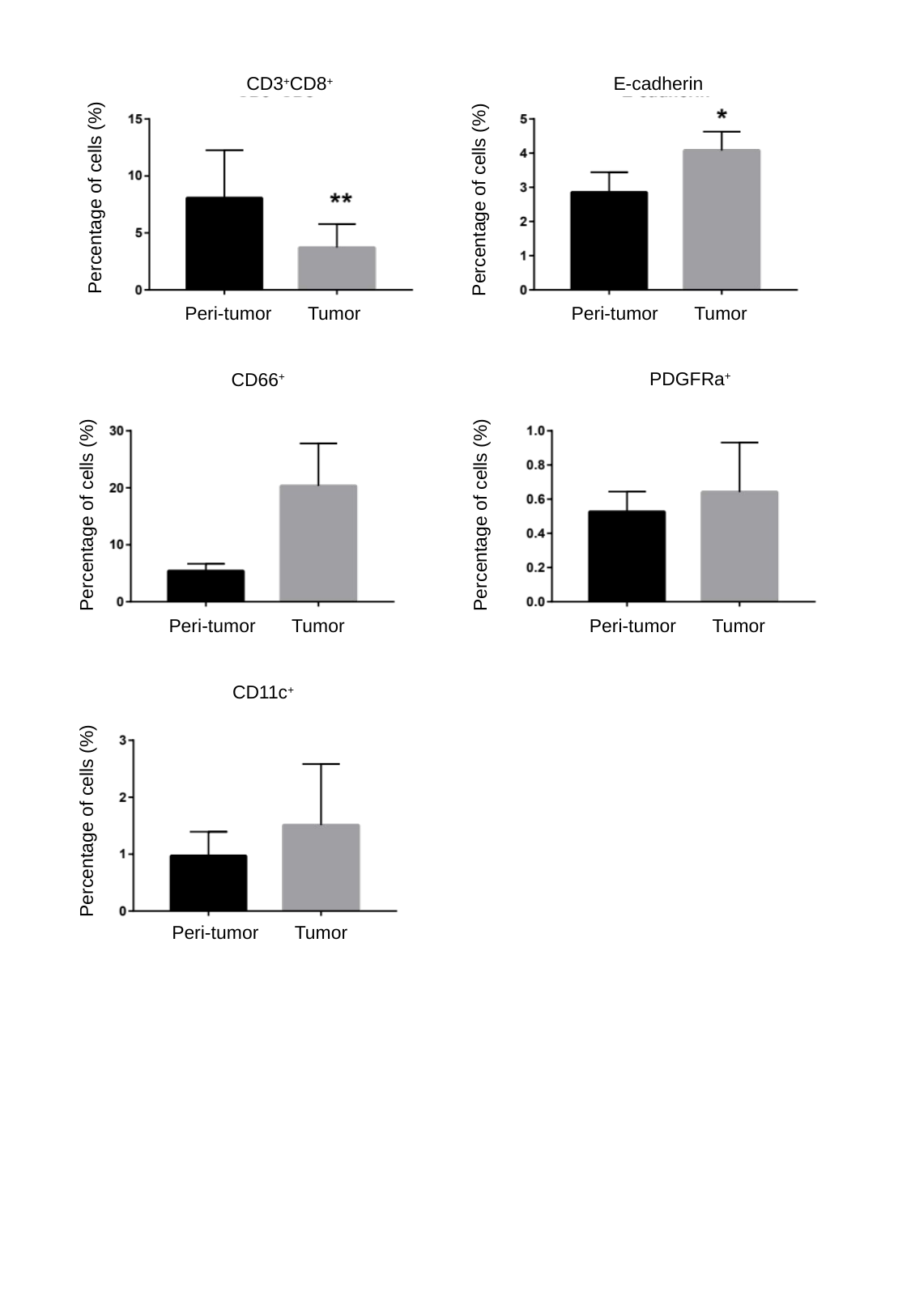

CD3+CD8+
E-cadherin
Percentage of cells (%)
Percentage of cells (%)
Peri-tumor
Tumor
Peri-tumor
Tumor
PDGFRa+
CD66+
Percentage of cells (%)
Percentage of cells (%)
Peri-tumor
Tumor
Peri-tumor
Tumor
CD11c+
Percentage of cells (%)
Peri-tumor
Tumor
