# Supplementary material for: Integrative Analysis of Genome, 3D Genome, and Transcriptome Alterations of Clinical Lung Cancer Samples
Source: Genomics Proteomics Bioinformatics. 2021 Jun 8;19(5):741–53. doi: 10.1016/j.gpb.2020.05.007 (PMC9170781; doi:10.1016/j.gpb.2020.05.007)
Supplement: Supplementary Figure S5 — Chromatin loop interactions identified from Hi-C data. A. and B. The number of overlapping and specific chromatin loops between samples identified by Fit-HiC (A) or HiCCUPUS (B). C. Aggregation peak analysis of chromatin loops identified by HiCCUPUS. D. Interaction heatmaps of a 1.2 Mb region on chromosome 1 (left) and chromosome 4 (right) of sample 6405N (blue, top right) and 6405T (red, bottom left). One normal specific loop (chr1:78.16-78.2 Mb with chr1:78.4-78.44 Mb) and one tumor specific loop (chr4:110.32-110.36 Mb with chr4:110.72-110.76 Mb) are highlighted. [file mmc5.pptx]

## Slide 1
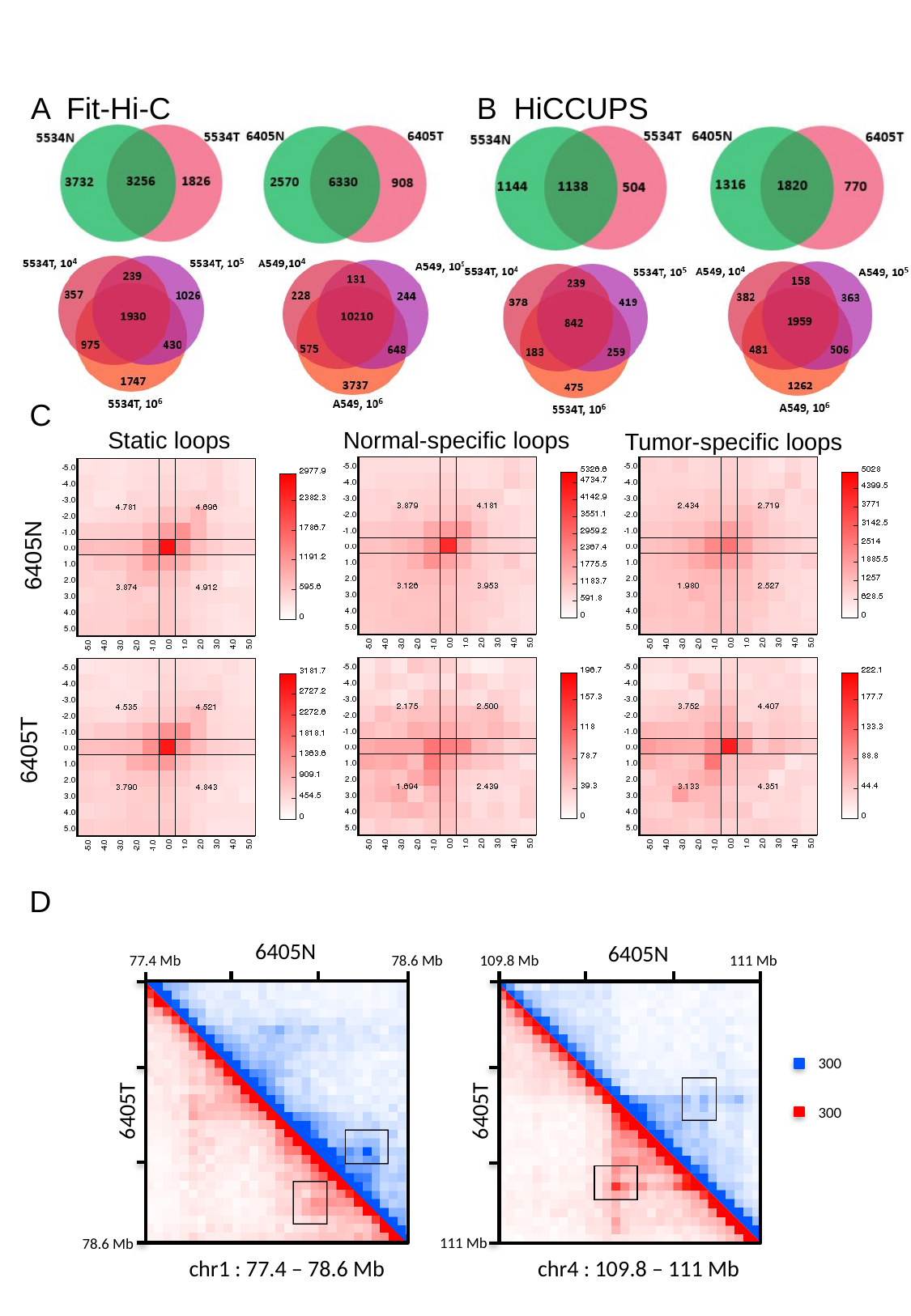

A Fit-Hi-C
B HiCCUPS
C
Static loops
Normal-specific loops
Tumor-specific loops
6405N
6405T
D
6405N
6405N
109.8 Mb
77.4 Mb
78.6 Mb
111 Mb
6405T
6405T
chr1 : 77.4 – 78.6 Mb
chr4 : 109.8 – 111 Mb
300
300
111 Mb
78.6 Mb
